# Innovation and constraints in cardiovascular pharmacotherapy

**DOI:** 10.1093/ehjcvp/pvag023

**Published:** 2026-05-19

**Authors:** Bianca Rocca

**Affiliations:** Department of Medicine and Surgery, LUM University, 70010 Casamassima (Ba), Italy

## Abstract

**Central figure:**
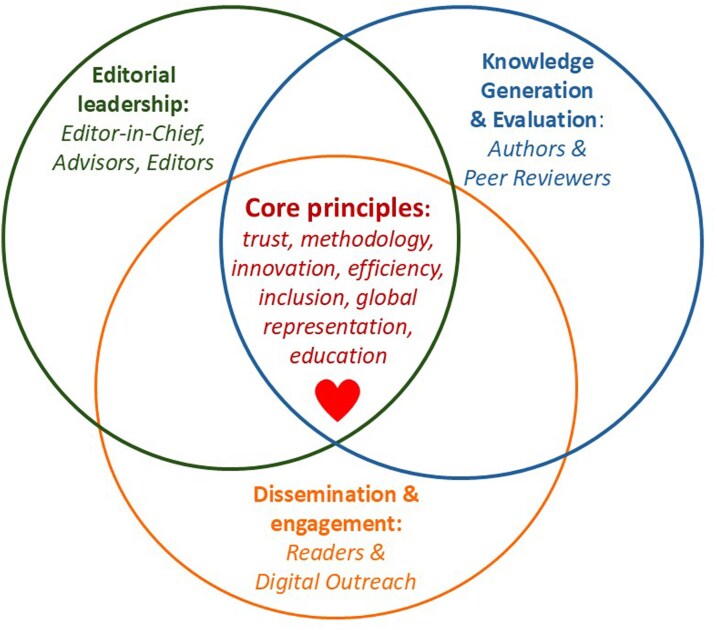
Conceptual model of the journal’s editorial and scientific ecosystem around its core principles.

Conceptual model of the journal’s editorial and scientific ecosystem around its core principles.


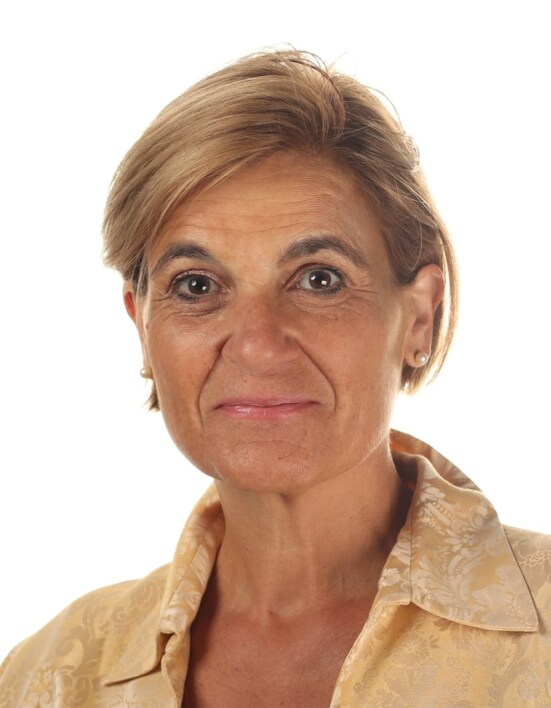


It is a privilege and a responsibility to assume the role of Editor-in-Chief of *the European Heart Journal—Cardiovascular Pharmacotherapy* at a time when cardiovascular therapeutics is evolving at an extraordinary pace and with increasing complexity. I wish to thank Professor Stefan Agewall for his leadership^1^ and the previous Editorial Board for their commitment to excellence, which have helped shape the Journal’s identity and success.

The new Editorial leadership represents continuity and renewal as the Journal enters its next phase. I am honoured to welcome the new Editorial Board Members,^2^ an international, multidisciplinary group of experts, to reflect the expanding scope of cardiovascular pharmacotherapy. I would like to express my sincere gratitude to all of them for their enthusiasm and commitment to the Journal, particularly through their dedication of time. Particularly, the representation of women scientists within the Board has considerably grown, reflecting the commitment to gender balance, equitable opportunities and inclusion. The multinational composition of the Board further broadens perspectives in cardiovascular research, strengthening attention to equitable access to innovative cardiovascular therapies and to the diverse socio-economic contexts across ESC member countries. A representative of the European Society of Cardiology (ESC) Patient Forum is part of the Board, to integrate patient perspectives into the Journal’s activities and reinforce our commitment to patient-centred cardiovascular pharmacotherapy. I would also like to express my gratitude to present and future peer reviewers, who remain cardinal to maintaining scientific standards. In alignment with the values of the ESC, we are committed to fostering the importance and value of diversity and open scientific dialogue, as keystones for scientific quality and impact.

Cardiovascular pharmacotherapy faces major, evolving challenges that require continuous attention. Increasing therapeutic complexity, often in the context of multimorbidity, ageing, necessary polypharmacy, and often resource limitations, requires knowledge and understanding of drugs (pharmacokinetics, pharmacodynamics, interactions), of their clinical efficacy and safety, of patient-centred management, and rational deprescribing strategies. The rapidly growing fields of (pharmaco)genomics, drug modelling, pharmacoepidemiology, pharmacovigilance, systems biology, and digital transformation necessitate careful integration of innovation with clinical applicability. The rapid expansion of real-world data offers new opportunities but also raises concerns regarding methodology, reproducibility, clinical relevance and interpretability.

My vision for the Journal is to further strengthen its role as a leading platform for rigorous, clinically meaningful, and methodologically sound research in cardiovascular pharmacotherapy, through the collective efforts of a great team composed of the Editorial Board, contributing Authors, Reviewers as well as Readers. The Journal will prioritize robust design, innovation, transparent and ethical reporting, and relevance for clinical practice and guideline development. We also aim to support early-career investigators through educational content on research methodology applied to clinical pharmacology, since education and mentorship will remain our mission. This integrated framework is summarized in the *Central figure*.

Within the Editorial Board,^2^ Advisors play a key role in shaping strategic initiatives, and supporting educational and scientific development. Strengthening training in clinical trial design, critical appraisal, and interpretation of evidence is a key priority. The knowledge and expertise of Associate Editors and of the wider Editorial Board will be central to ensuring rigorous, timely, and consistent review processes and editorial decision-making. We will expand digital outreach to foster engagement, dissemination and discussion of high-impact research, and I would like to thank our social media Editors, whose creativity and dedication are supporting this effort.

In this issue, we feature contributions that mirror the breadth and dialectical complexity of research in cardiovascular pharmacology.

‘*Why does research integrity affect us all?’*^3^ by Christian Funck-Brentano and Ghislaine Filliatreau underscores the fundamental importance of methodological rigour and ethical responsibility in biomedical research. The reliability of clinical evidence, innovation, regulatory decisions and guideline implementation ultimately depends on the integrity of the entire scientific process. Research integrity is fundamental for the scientific community, for the entire society, for public trust in science and for investments in pharmacological research and innovation.

Two contributions in the issue illustrate the dynamic lifecycle of cardiovascular therapeutics. ‘*Targeted Anticoagulant Reversal, Unintended Consequences: Lessons From Andexanet Alfa*’^4^ by Jerrold H. Levy and Joseph R. Shaw, provides a timely and instructive example of the complex journey of drug development and evaluation. The Authors carefully examine the trajectory of the antidote andexanet alfa, from small trials with surrogate endpoints, accelerated approval, to subsequent safety concerns, until the final request to the FDA to voluntarily withdraw the drug from the US market. This experience underscores the relevance of many steps: pre-clinical and clinical development, trial design, validation and interpretation of surrogate markers, tight post-approval evaluation with continuous assessment of benefit-risk profiles. In parallel, the comprehensive overview of cardiovascular pharmacotherapy in 2025 by Tamargo *et al*.^5^ summarizes the year’s new drug applications and approvals, label extensions, and randomized clinical trials, including both positive and neutral or negative trials. Collectively, these data illustrate a field in continuous and rapid evolution, with an expanding range of therapeutic mechanisms and strategies across cardiovascular disease. At the same time, they highlight that final clinical translation remains heterogeneous, with not all pharmacological advances demonstrating clear improvements in patient outcomes. This duality underscores the ongoing challenges in cardiovascular drug development.

With respect to evidence generation, two studies in this issue focus on clinical trial data. Morten Würtz *et al*.^6^ performed an interesting *post hoc* analysis, using data from the cohort of the COMPASS trial. By applying the 2024 ESC chronic coronary syndrome^7^ criteria for risk stratification, this study showed that these criteria provided only modest separation of low vs. high ischaemic risk at baseline (C-index 0.52) and could not discriminate subgroups with differential net benefit from dual pathway inhibition. The pre-specified Bayesian reanalysis of the FINEARTS-HF trial by Alasdair D. Henderson *et al*.^8^ confirms the frequentist findings of the reduction in cardiovascular death and heart failure-related events with finerenone in patients with heart failure and mildly reduced or preserved ejection fraction. By incorporating prior evidence, the analysis provides probabilistic estimates of benefit, including an approximately 90% probability of at least a 10% reduction in the primary outcome, with lower but still substantial probabilities for mortality. Safety analyses confirm an increased probability of hyperkalaemia. Overall, the study illustrates how Bayesian methods may complement and refine conventional analyses by expressing both efficacy and safety in probabilistic terms.

Altogether, these contributions and others published in this issue^9^ converge towards a central theme: cardiovascular pharmacotherapy is defined by therapeutic innovation, the strength and integrity of its evidence base, the robustness of its translational and regulatory foundations, and the increasing value of complementary analytical approaches that refine the interpretation of clinical trial data.

I invite our readers, authors, and reviewers to engage with us in this shared journey.

## Data Availability

No data were generated by this editorial.

## References

[1] Dobrev D, Kaski JC, Rocca B, Drexel H, BS L, Niessner A. A farewell to professor Stefan Agewall from the ESC working group on cardiovascular pharmacotherapy. Eur Heart J Cardiovasc Pharmacother 2026;12:57. 41757726 10.1093/ehjcvp/pvag004 PMC12946959

[2] https://academic.oup.com/ehjcvp/pages/Editorial_Board. Date accessed 15 April 2026.

[3] Funck-Brentano C, Filliatreau G. Why does research integrity affect us all? Eur Heart J Cardiovasc Pharmacother 2026;12:142–143. 10.1093/ehjcvp/pvag015 PMC13185739 41816923

[4] Levy JH, Shaw JR. Targeted anticoagulant reversal, unintended consequences: lessons from andexanet alfa. Eur Heart J Cardiovasc Pharmacother 2026;12:144–146. 10.1093/ehjcvp/pvag014 PMC13185740 41821513

[5] Tamargo J, Agewall S, Ambrosio G, Borghi C, Cerbai E, Dan GA, Drexel H, Ferdinandy P, Grove EL, Klingenberg R, Morais J, Parker W, Rocca B, Sulzgruber P, Semb AG, Sossalla S, Kaski JC, Dobrev D. Cardiovascular pharmacotherapy in year in 2025. Eur Heart J Cardiovasc Pharmacother 2026;12:147–166. 10.1093/ehjcvp/pvag016 PMC13185746 41935384

[6] Würtz M, Olesen KKW, Yi Q, Eikelboom JW, Maeng M. Net clinical benefit of extended dual pathway inhibition in chronic coronary syndrome as classified by the 2024 ESC criteria: a COMPASS substudy. Eur Heart J Cardiovasc Pharmacother 2026;12:167–175. 10.1093/ehjcvp/pvag008 PMC13185743 41614385

[7] Vrints C, Andreotti F, Koskinas KC, Rossello X, Adamo M, Ainslie J, Banning AP, Budaj A, Buechel RR, Chiariello GA. 2024 ESC guidelines for the management of chronic coronary syndromes. Eur Heart J 2024;45:3415–3537. 39210710 10.1093/eurheartj/ehae177

[8] Henderson AD, Docherty KF, Talebi A, Kondo T, Petrie MC, Claggett BL, Desai AS, Vaduganathan M, Atherton JJ, Bauersachs J, Schou M, Verma S, Lam CSP, Pitt B, Senni M, Shah SJ, Voors AA, Zannad F, Brinker M, Amarante F, Rohwedder K, Lay-Flurrie J, Solomon SD, McMurray JJV, Jhund PS. A Bayesian analysis of finerenone in heart failure with mildly reduced and preserved ejection fraction: a pre-specified analysis of FINEARTS-HF. Eur Heart J Cardiovasc Pharmacother 2026;12:176–185. 10.1093/ehjcvp/pvag010 PMC13185745 41697954

[9] Loutati R, Copeland V, Elimeleh S, Hochstein D, Faierstein K, Milwidsky A, Ben-Zekry S, Segev A, Kuperstein R, Maor E. Heart failure and tricuspid regurgitation: the role of SGLT2 inhibitors in improving outcomes. Eur Heart J Cardiovasc Pharmacother 2026;12:186–197. 10.1093/ehjcvp/pvag018 PMC13185744 41906747

